# Supplementary material for: Integration of Candida albicans-induced single-cell gene expression data and secretory protein concentrations reveal genetic regulators of inflammation
Source: Front Immunol. 2023 Feb 14;14:1069379. doi: 10.3389/fimmu.2023.1069379 (PMC9972217; doi:10.3389/fimmu.2023.1069379)
Supplement: Supplementary file 1 [file DataSheet_1.docx]

**Figure S1: Quality control and pre-processing of protein abundances**

**(A)** Dot plot depicting all proteins measured by the Olink inflammatory panel (horizontal axis) against missing frequency (vertical axis). Missingness per protein denotes samples measured below the detection limit of the assay. The horizontal dashed red line represents 15% missingness threshold used to exclude proteins with high missingness. (**B)** Distribution of proteins using Normal Protein expression (NPx) in Log2 scale (**C**) Distribution of proteins using inverse rank normalized values. Figures were generated with the “ggplot” function.

**Figure S2: Summary of univariate pQTL mapping results**

**
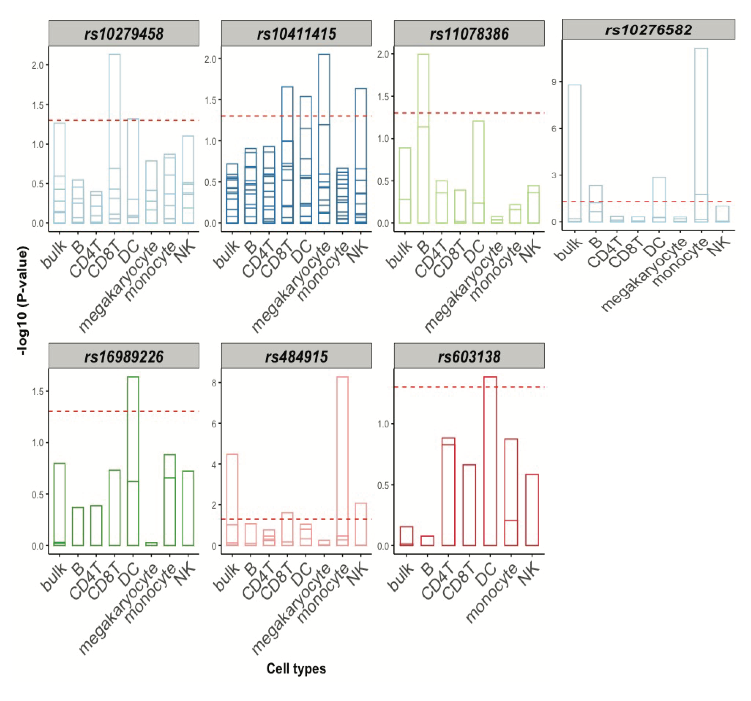
**

**Figure S3: Top protein QTL variants show association with cis-eQTLs after 24h candida albicans stimulation**

The horizontal red dashed line corresponds to 0.05 P-value, FDR corrected. Each strip in the bar represents a gene associated with the corresponding SNPs. Barplots were generated with the ggplot in R.

**Figure S4: Trans-pQTL associations and their protein-protein interactions**

**(A** – **C**) Regional association plots generated with LocusZoom for regions around the top pQTL variants (purple diamond) for Flt3L, LIF and EN-RAGE respectively. Genomic positions are indicated on the x-axes and the annotated genes in the trans loci are displayed above the x-axes. (**D** – **F**) Network of genes encoding for the protein interactors. The octagon-shaped blue nodes are the corresponding genes of the tested proteins in (**A**-**C**) and the gray nodes represent the genes that encoded for the proteins interacting with the tested proteins. The numbers on the edges represent the confidence score of the interactions which is computed based on the number of studies or experimental techniques supporting the interactions detected and it ranges between 0 and 1.

**Figure S5: Trans-pQTL associations and their protein-protein interactions**

**(A** – **C**) Regional association plots generated with LocusZoom for regions around the top pQTL variants (purple diamond) for Flt3L, LIF and EN-RAGE respectively. Genomic positions are indicated on the x-axes and the annotated genes in the trans loci are displayed above the x-axes. (**D** – **F)** Network of genes encoding for the protein interactors. The octagon-shaped blue nodes are the corresponding genes of the tested proteins in (**A**-**C**) and the gray nodes represent the genes that encoded for the proteins interacting with the tested proteins. The numbers on the edges represent the confidence score of the interactions which is computed based on the number of studies or experimental techniques supporting the interactions detected and it ranges between 0 and 1.

**Figure S6: Summary of multivariate QTL mapping results**

**(A)** Manhattan plot of the pQTL mapping in the 500FG cohort. (**B)** The quantile-quantile (QQ) plot for the association results in the 500FG (**C**) Manhattan plot of the pQTL mapping in the 1M-scBloodNL cohort. (**D**) The quantile-quantile (QQ) plot for the association results in the 1MscBloodNL cohort. The genomic inflation factor is denoted by lambda (λ).
